# Supplementary material for: Language structure is influenced by the number of speakers but seemingly not by the proportion of non-native speakers
Source: R Soc Open Sci. 2019 Feb 27;6(2):181274. doi: 10.1098/rsos.181274 (PMC6408393; doi:10.1098/rsos.181274)
Supplement: Electronic supplementary material [file rsos181274supp1.docx]

**Supplementary material for “Language structure is influenced by the number of speakers but seemingly not by the proportion of non-native speakers”**

**Alexander Koplenig^1^**

**1** Institute for the German Language (IDS), Mannheim, Germany

# Section 1. Linear mixed model analysis

The linear mixed model analysis (cf. Table 1) demonstrates that, in all cases but one, neither the *vehicularity* variable nor the interaction between the estimated speaker population size (logged) and *vehicularity* is statistically significant. The only exception is the model in row 10, where morphological complexity is estimated with fixed effects for population size, *vehicularity* and their interaction in languages with available *WALS* information for at least 6 features. However, statistical significance is only achieved without the *Bonferroni* adjustment. In addition, this model does not support the linguistic niche hypothesis, either, as the direction of the interaction points in the positive direction and thus indicates that *vehicular* languages (i.e. languages with a significant amount of *L2* speakers) become more morphologically complex if the population size is increased. In addition, the model does not contain any random effects in order to control for the non-independence of data-points due to genetic and areal relationships between languages [1]. In the corresponding model with included random effects (row 12, 14 & 16), neither the coefficient for the *vehicularity* variable nor the interaction reaches statistical significance. It is worth pointing out that in correspondence with the other results presented in this paper, population size significantly predicts morphological and information theoretic complexity in most models.

Table 1: Results of the linear mixed model analysis.

| Row | Dependent variable | Control variable (fixed) | Intercepts (random) | Slopes (random) | β_  logPop | β_ vehicularity | β_ interaction | N | NF |
| --- | --- | --- | --- | --- | --- | --- | --- | --- | --- |
| 1 | Morphological complexity | Population, vehicularity |  |  | -0.008* | -0.061 |  | 1,581 | 1 |
| 2 | Morphological complexity | Population, vehicularity, interaction |  |  | -0.009** | -0.224 | 0.012 | 1,581 | 1 |
| 3 | Morphological complexity | Population, vehicularity | Families |  | -0.008* | -0.058 |  | 1,581 | 1 |
| 4 | Morphological complexity | Population, vehicularity, interaction | Families |  | -0.008* | -0.085 | 0.002 | 1,581 | 1 |
| 5 | Morphological complexity | Population, vehicularity | Families | Families | -0.005 | -0.066 |  | 1,172 | 1 |
| 6 | Morphological complexity | Population, vehicularity, interaction | Families | Families | -0.006 | -0.209 | 0.010 | 1,074 | 1 |
| 7 | Morphological complexity | Population, vehicularity | Families & areas |  | -0.009* | -0.039 |  | 1,512 | 1 |
| 8 | Morphological complexity | Population, vehicularity, interaction | Families & areas |  | -0.010* | -0.058 | 0.001 | 1,512 | 1 |
| 9 | Morphological complexity | Population, vehicularity |  |  | -0.008* | -0.040 |  | 862 | 6 |
| 10 | Morphological complexity | Population, vehicularity, interaction |  |  | -0.011** | -0.365* | 0.023* | 862 | 6 |
| 11 | Morphological complexity | Population, vehicularity | Families |  | -0.007 | -0.036 |  | 862 | 6 |
| 12 | Morphological complexity | Population, vehicularity, interaction | Families |  | -0.008* | -0.139 | 0.008 | 862 | 6 |
| 13 | Morphological complexity | Population, vehicularity | Families | Families | -0.005 | -0.038 |  | 582 | 6 |
| 14 | Morphological complexity | Population, vehicularity, interaction | Families | Families | -0.009 | -0.326 | 0.020 | 510 | 6 |
| 15 | Morphological complexity | Population, vehicularity | Families & areas |  | -0.008* | -0.032 |  | 821 | 6 |
| 16 | Morphological complexity | Population, vehicularity, interaction | Families & areas |  | -0.009* | -0.119 | 0.006 | 821 | 6 |
| 17 | Entropy rate | Population, vehicularity |  |  | 0.036** | 0.036 |  | 1,088 |  |
| 18 | Entropy rate | Population, vehicularity, interaction |  |  | 0.035** | -0.069 | 0.008 | 1,088 |  |
| 19 | Entropy rate | Population, vehicularity | Families |  | 0.021** | 0.011 |  | 1,088 |  |
| 20 | Entropy rate | Population, vehicularity, interaction | Families |  | 0.020** | -0.092 | 0.008 | 1,088 |  |
| 21 | Entropy rate | Population, vehicularity | Families | Families | 0.021** | -0.006 |  | 842 |  |
| 22 | Entropy rate | Population, vehicularity, interaction | Families | Families | 0.023** | -0.000 | -0.000 | 731 |  |
| 23 | Entropy rate | Population, vehicularity | Families & areas |  | 0.025** | -0.003 | . | 719 |  |
| 24 | Entropy rate | Population, vehicularity, interaction | Families & areas |  | 0.024** | -0.033 | 0.002 | 719 |  |

1st column: Row number (for reference). 2nd column: dependent variable. 3rd column: fixed effects. 4th column: random intercepts. 5th column: random slopes 6th-8th column: *β* coefficient of the corresponding predictor. 9th column: number of available languages. 10th column: number of included *WALS* features/chapters (if relevant). *NB*.: The population size is logged in all models. Models with random slopes (for population size and the interaction variable) only include language families with at least (i) 20 data points for the models without interaction and (ii) 30 data points for the models with interaction as suggested by [1]. Values are rounded for illustration purposes only. One asterisks (*) indicates that the corresponding coefficient passed the permutation test at *p* < .01. Two asterisks (**) denote statistical significance after the Bonferroni adjustment [*m* = 24].

# Section 2. Results of the permutation test with the unigram entropy as the dependent variable

[2] present empirical evidence that suggests a negative relationship between lexical diversity and the proportion of *L2* speakers. As for the sample used by [3] mentioned in the introduction of the main text, it is not clear if the sample of [2] is unbiased, because (i) it only comprises 91 languages; (ii) compared to the median estimated speaker population size of 7,000 for the roughly 7,000 languages listed by the *Ethnologue* [4], the median estimated speaker population size in the sample of [2] is 9,648,300; (iii) all 91 languages have an estimated proportion of *L2* speakers that is greater than zero with a median estimate of roughly 33%; (iv) there is no (Spearman) correlation between the estimated speaker population size and the estimated proportion of *L2* speakers (*r =* 0.057).

The results of a permutation test are presented below.

In [2], the average information content of word types is used as one measure of lexical diversity. For a distribution of *i* = 1, 2, …, *k* different words with a token frequency of *f_i_*, it can be defined as:

$H_{word}=-\sum_{i=1}^{k} \frac{f_{i}}{\sum_{i=1}^{k} f_{i}}*{log}_{2}(\frac{f_{i}}{\sum_{i=1}^{k} f_{i}})$ [1]

Higher entropies of word frequencies are indicative of higher lexical diversities [2]. Here, information made available by [5] regarding word entropy estimates for 1,080 languages based on the *Parallel Bible Corpus* [6] is used. Table 2 presents the results of the permutation tests with the word entropy as the dependent variable. The results demonstrate that *vehicularity* only significantly predicts lexical diversity in a model without control for potential confounding variables. It is worth pointing out that the direction of the relationship is the opposite of the results presented by [2] as it suggests that *vehicular* language, i.e. languages that tend to have a significant number of *L2* users according to the *Ethnologue*, tend to have higher lexical diversities. However, in all models with fixed control for the estimated speakers population size (logged) and random controls for language families and areas, the coefficient of determination for *vehicularity* is below 1% and does not reach significance (at *p* < .01). These results question the idea that large proportions of *L2* speakers affect the lexical diversity of languages.

Table 2: Results of the permutation test with the unigram entropy as dependent variable

| Depvar | Control_fixed | Control_random | R2 | Direction | N |
| --- | --- | --- | --- | --- | --- |
| Unigram entropy | no control |  | 1.77** | + | 1,080 |
|  | Population size |  | 0.13 | + | 1,080 |
|  | Population size | Families | 0.00 | - | 1,080 |
|  | Population size | Areas | 0.28 | - | 718 |
|  | Population size | Families & Areas | 0.27 | - | 718 |
|  | Population size | Families (intercepts & slopes) | 0.14 | - | 900 |
|  | Population size | Areas (intercepts & slopes) | 0.49 | - | 691 |

Cf. Table 1 (main text) for a description of the columns. Here, *m* = 10 for the *Bonferroni* adjustment.

# Section 3. Between-families and between-areas tests

Table 3 shows that the associational pattern found in Table 2 in the main part of the paper also holds for the Monte Carlo simulations, both for language families and for geographical language areas. In both cases:

1. The strongest absolute correlation is found between the entropy rate and the speaker population size.
2. There is no noteworthy negative Spearman correlation between morphological complexity and the relative proportion of *L2* speakers (especially when the effect of the population size is partialled out).
3. The Spearman correlations between morphological complexity and the entropy rate both seem to be virtually non-existent.

Table 3: Between-families and between-areas tests (Monte Carlo simulations).

| Row | *r*_v1v2_ | *N_r_* | *pr*_v1v2z_ | *N_r_* | *N_F_* |
| --- | --- | --- | --- | --- | --- |
| Family |  |  |  |  |  |
| 1 | v1: Speaker population size \| v2: L2 proportion |  |  |  |  |
|  | 0.208 | 50 |  |  | 1 |
| 2 | v1: Morphological complexity \| v2: L2 proportion |  | z: Speaker population size |  |  |
|  | -0.105 | 45 | -0.079 | 45 | 1 |
|  | -0.057 | 27 | -0.019 | 27 | 6 |
| 3 | v1: Morphological complexity \| v2: Speaker population size |  | z: L2 proportion |  |  |
|  | -0.130 | 47 | -0.112 | 45 | 1 |
|  | -0.135 | 27 | -0.121 | 27 | 6 |
| 4 | v1: Entropy rate \| v2: L2 proportion |  | z: Speaker population size |  |  |
|  | 0.296 | 28 | 0.130 | 28 | 1 |
| 5 | v1: Entropy rate \| v2: Speaker population size |  | z: L2 proportion |  |  |
|  | 0.516 | 31 | 0.330 | 28 | 1 |
| 6 | v1: Morphological complexity \| v2: Entropy rate |  |  |  |  |
|  | -0.075 | 24 |  |  | 1 |
|  | 0.083 | 17 |  |  | 6 |
| Area |  |  |  |  |  |
| 1 | v1: Speaker population size \| v2: L2 proportion |  |  |  |  |
|  | 0.309 | 24 |  |  | 1 |
| 2 | v1: Morphological complexity \| v2: L2 proportion |  | z: Speaker population size |  |  |
|  | -0.131 | 24 | -0.086 | 24 | 1 |
|  | -0.160 | 24 | -0.093 | 24 | 6 |
| 3 | v1: Morphological complexity \| v2: Speaker population size |  | z: L2 proportion |  |  |
|  | -0.177 | 24 | -0.120 | 24 | 1 |
|  | -0.208 | 24 | -0.153 | 24 | 6 |
| 4 | v1: Entropy rate \| v2: L2 proportion |  | z: Speaker population size |  |  |
|  | 0.426 | 18 | 0.069 | 18 | 1 |
| 5 | v1: Entropy rate \| v2: Speaker population size |  | z: L2 proportion |  |  |
|  | 0.640 | 18 | 0.445 | 18 | 1 |
| 6 | v1: Morphological complexity \| v2: Entropy rate |  |  |  |  |
|  | -0.062 | 17 |  |  | 1 |
|  | 0.039 | 16 |  |  | 6 |

Cf. Table 2 (main text) for a description of the columns. Values in column 2 and 4 denote *z*-transformed average Spearman correlations and z-transformed average Spearman part correlations over 100,000 repetitions. Per repetition, one observation is randomly drawn from each language family/area. *NB*.: no additional permutation tests have been conducted for this kind of Monte Carlo simulation.

# Section 4. Within-families and within-areas correlation analysis

To select language families and areas that are suitable for analysis, all families and areas were excluded, for which there was no or insufficient variation on the variable “proportion of *L2* speakers”. To show that this criterion is necessary, take for example the Otomanguean language family that consists of 77 languages. However, all respective languages have a proportion of *L2* speakers that is equal to 0. Obviously, including such a variable in the correlation analysis would not make sense, since a correlation between a variable and a constant is undefined.

The six largest language families and language areas were selected. While, due to noise in the data [1], it is unlikely that the results for the within-families and within-areas are the same for all groups, the general trend remains comparable, as Table 4 demonstrates:

1. There is a positive Spearman correlation between the speaker population size and *L2* proportion for all 6 language families (significant in 5 cases [*Bonferroni* adjusted in 3 cases]) and for all 6 language areas (significant in 5 cases [*Bonferroni* adjusted in 3 cases]).
2. There is a positive Spearman correlation between the entropy rate and the speaker population size for 5 of 6 language families (significant in 3 cases [*Bonferroni* adjusted in 1 case]) and for all language areas (significant in 3 cases [*Bonferroni* adjusted in 1 case]).
3. There is a negative Spearman correlation between morphological complexity and the *L2* proportion after partialling out the effect of the speaker population size for 3 of 6 language families and for 4 of 6 language areas. However only in one case, the coefficient passes the permutation test [*Bonferroni* adjusted in 0 cases]. In 9 of those cases, the part correlation is either positive or the absolute value of the correlation coefficient is below 0.1.
4. There is positive Spearman correlation between the entropy rate and *L2* proportion after partialling out the effect of the speaker population for 3 of 6 language families and for 1 of 6 language areas. None of those correlation coefficients pass the permutation test.

Table 4: Within-families and within-areas correlation analysis.

| Row | *r*_v1v2_ | *N_r_* | *pr*_v1v2z_ | *N_r_* | *N_F_* |
| --- | --- | --- | --- | --- | --- |
| Family: Afro-Asiatic |  |  |  |  |  |
| 1 | v1: Speaker population size \| v2: L2 proportion |  |  |  |  |
|  | 0.239* | 97 |  |  |  |
| 2 | v1: Morphological complexity \| v2: L2 proportion |  | z: Speaker population size |  |  |
|  | 0.139 | 83 | 0.160 | 83 | 1 |
| 3 | v1: Morphological complexity \| v2: Speaker population size |  | z: L2 proportion |  |  |
|  | -0.129 | 92 | -0.130 | 83 | 1 |
| 4 | v1: Entropy rate \| v2: L2 proportion |  | z: Speaker population size |  |  |
|  | 0.382 | 35 | 0.101 | 35 |  |
| 5 | v1: Entropy rate \| v2: Speaker population size |  | z: L2 proportion |  |  |
|  | 0.597** | 39 | 0.512** | 35 |  |
| 6 | v1: Morphological complexity \| v2: Entropy rate |  |  |  |  |
|  | 0.334 | 25 |  |  | 1 |
| Family: Altaic |  |  |  |  |  |
| 1 | v1: Speaker population size \| v2: L2 proportion |  |  |  |  |
|  | 0.416* | 30 |  |  |  |
| 2 | v1: Morphological complexity \| v2: L2 proportion |  | z: Speaker population size |  |  |
|  | -0.054 | 28 | -0.130 | 28 | 1 |
| 3 | v1: Morphological complexity \| v2: Speaker population size |  | z: L2 proportion |  |  |
|  | 0.085 | 39 | 0.189 | 28 | 1 |
| 4 | v1: Entropy rate \| v2: L2 proportion |  | z: Speaker population size |  |  |
|  | 0.283 | 11 | -0.055 | 11 |  |
| 5 | v1: Entropy rate \| v2: Speaker population size |  | z: L2 proportion |  |  |
|  | 0.284 | 19 | 0.405 | 11 |  |
| 6 | v1: Morphological complexity \| v2: Entropy rate |  |  |  |  |
|  | -0.063 | 17 |  |  | 1 |
| Family: Austronesian |  |  |  |  |  |
| 1 | v1: Speaker population size \| v2: L2 proportion |  |  |  |  |
|  | 0.147* | 285 |  |  |  |
| 2 | v1: Morphological complexity \| v2: L2 proportion |  | z: Speaker population size |  |  |
|  | -0.092 | 165 | -0.083 | 165 | 1 |
| 3 | v1: Morphological complexity \| v2: Speaker population size |  | z: L2 proportion |  |  |
|  | -0.018 | 191 | -0.055 | 165 | 1 |
| 4 | v1: Entropy rate \| v2: L2 proportion |  | z: Speaker population size |  |  |
|  | 0.104 | 173 | 0.061 | 173 |  |
| 5 | v1: Entropy rate \| v2: Speaker population size |  | z: L2 proportion |  |  |
|  | 0.287** | 190 | 0.212* | 173 |  |
| 6 | v1: Morphological complexity \| v2: Entropy rate |  |  |  |  |
|  | 0.115 | 68 |  |  | 1 |
| Family: Indo-European |  |  |  |  |  |
| 1 | v1: Speaker population size \| v2: L2 proportion |  |  |  |  |
|  | 0.675** | 64 |  |  |  |
| 2 | v1: Morphological complexity \| v2: L2 proportion |  | z: Speaker population size |  |  |
|  | -0.492** | 56 | -0.399* | 56 | 1 |
| 3 | v1: Morphological complexity \| v2: Speaker population size |  | z: L2 proportion |  |  |
|  | -0.168 | 87 | 0.052 | 56 | 1 |
| 4 | v1: Entropy rate \| v2: L2 proportion |  | z: Speaker population size |  |  |
|  | -0.029 | 32 | -0.103 | 32 |  |
| 5 | v1: Entropy rate \| v2: Speaker population size |  | z: L2 proportion |  |  |
|  | 0.164 | 55 | 0.156 | 32 |  |
| 6 | v1: Morphological complexity \| v2: Entropy rate |  |  |  |  |
|  | 0.442* | 46 |  |  | 1 |
| Family: Niger-Congo |  |  |  |  |  |
| 1 | v1: Speaker population size \| v2: L2 proportion |  |  |  |  |
|  | 0.426** | 339 |  |  |  |
| 2 | v1: Morphological complexity \| v2: L2 proportion |  | z: Speaker population size |  |  |
|  | 0.036 | 218 | 0.026 | 218 | 1 |
| 3 | v1: Morphological complexity \| v2: Speaker population size |  | z: L2 proportion |  |  |
|  | 0.028 | 235 | 0.011 | 218 | 1 |
| 4 | v1: Entropy rate \| v2: L2 proportion |  | z: Speaker population size |  |  |
|  | 0.135 | 185 | 0.041 | 185 |  |
| 5 | v1: Entropy rate \| v2: Speaker population size |  | z: L2 proportion |  |  |
|  | 0.257** | 204 | 0.162 | 185 |  |
| 6 | v1: Morphological complexity \| v2: Entropy rate |  |  |  |  |
|  | 0.392** | 75 |  |  | 1 |
| Family: Sino-Tibetan |  |  |  |  |  |
| 1 | v1: Speaker population size \| v2: L2 proportion |  |  |  |  |
|  | 0.186 | 101 |  |  |  |
| 2 | v1: Morphological complexity \| v2: L2 proportion |  | z: Speaker population size |  |  |
|  | -0.030 | 90 | 0.007 | 90 | 1 |
| 3 | v1: Morphological complexity \| v2: Speaker population size |  | z: L2 proportion |  |  |
|  | -0.264* | 99 | -0.174 | 90 | 1 |
| 4 | v1: Entropy rate \| v2: L2 proportion |  | z: Speaker population size |  |  |
|  | -0.039 | 24 | -0.014 | 24 |  |
| 5 | v1: Entropy rate \| v2: Speaker population size |  | z: L2 proportion |  |  |
|  | -0.013 | 30 | -0.024 | 24 |  |
| 6 | v1: Morphological complexity \| v2: Entropy rate |  |  |  |  |
|  | 0.238 | 17 |  |  | 1 |
| Area: African-Savannah |  |  |  |  |  |
| 1 | v1: Speaker population size \| v2: L2 proportion |  |  |  |  |
|  | 0.312** | 229 |  |  |  |
| 2 | v1: Morphological complexity \| v2: L2 proportion |  | z: Speaker population size |  |  |
|  | -0.025 | 192 | 0.050 | 192 | 1 |
| 3 | v1: Morphological complexity \| v2: Speaker population size |  | z: L2 proportion |  |  |
|  | -0.203* | 209 | -0.227** | 192 | 1 |
| 4 | v1: Entropy rate \| v2: L2 proportion |  | z: Speaker population size |  |  |
|  | 0.116 | 95 | 0.003 | 95 |  |
| 5 | v1: Entropy rate \| v2: Speaker population size |  | z: L2 proportion |  |  |
|  | 0.326** | 108 | 0.256* | 95 |  |
| 6 | v1: Morphological complexity \| v2: Entropy rate |  |  |  |  |
|  | 0.283* | 67 |  |  | 1 |
| Area: Europe |  |  |  |  |  |
| 1 | v1: Speaker population size \| v2: L2 proportion |  |  |  |  |
|  | 0.538* | 24 |  |  |  |
| 2 | v1: Morphological complexity \| v2: L2 proportion |  | z: Speaker population size |  |  |
|  | -0.387 | 20 | -0.393 | 20 | 1 |
| 3 | v1: Morphological complexity \| v2: Speaker population size |  | z: L2 proportion |  |  |
|  | 0.054 | 37 | 0.089 | 20 | 1 |
| 4 | v1: Entropy rate \| v2: L2 proportion |  | z: Speaker population size |  |  |
|  | -0.102 | 20 | -0.068 | 20 |  |
| 5 | v1: Entropy rate \| v2: Speaker population size |  | z: L2 proportion |  |  |
|  | 0.105 | 34 | -0.032 | 20 |  |
| 6 | v1: Morphological complexity \| v2: Entropy rate |  |  |  |  |
|  | 0.443* | 30 |  |  | 1 |
| Area: Greater-Abyssinia |  |  |  |  |  |
| 1 | v1: Speaker population size \| v2: L2 proportion |  |  |  |  |
|  | 0.075 | 39 |  |  |  |
| 2 | v1: Morphological complexity \| v2: L2 proportion |  | z: Speaker population size |  |  |
|  | 0.063 | 37 | 0.073 | 37 | 1 |
| 3 | v1: Morphological complexity \| v2: Speaker population size |  | z: L2 proportion |  |  |
|  | -0.224 | 37 | -0.227 | 37 | 1 |
| 4 | v1: Entropy rate \| v2: L2 proportion |  | z: Speaker population size |  |  |
|  | -0.200 | 10 | -0.140 | 10 |  |
| 5 | v1: Entropy rate \| v2: Speaker population size |  | z: L2 proportion |  |  |
|  | 0.243 | 10 | 0.197 | 10 |  |
| 6 | v1: Morphological complexity \| v2: Entropy rate |  |  |  |  |
|  | 0.452 | 8 |  |  | 1 |
| Area: Indic |  |  |  |  |  |
| 1 | v1: Speaker population size \| v2: L2 proportion |  |  |  |  |
|  | 0.400** | 95 |  |  |  |
| 2 | v1: Morphological complexity \| v2: L2 proportion |  | z: Speaker population size |  |  |
|  | -0.150 | 94 | -0.035 | 94 | 1 |
| 3 | v1: Morphological complexity \| v2: Speaker population size |  | z: L2 proportion |  |  |
|  | -0.334** | 104 | -0.267* | 94 | 1 |
| 4 | v1: Entropy rate \| v2: L2 proportion |  | z: Speaker population size |  |  |
|  | 0.351 | 15 | -0.041 | 15 |  |
| 5 | v1: Entropy rate \| v2: Speaker population size |  | z: L2 proportion |  |  |
|  | 0.556* | 20 | 0.475 | 15 |  |
| 6 | v1: Morphological complexity \| v2: Entropy rate |  |  |  |  |
|  | -0.284 | 19 |  |  | 1 |
| Area: Oceania |  |  |  |  |  |
| 1 | v1: Speaker population size \| v2: L2 proportion |  |  |  |  |
|  | 0.219* | 142 |  |  |  |
| 2 | v1: Morphological complexity \| v2: L2 proportion |  | z: Speaker population size |  |  |
|  | -0.048 | 115 | -0.075 | 115 | 1 |
| 3 | v1: Morphological complexity \| v2: Speaker population size |  | z: L2 proportion |  |  |
|  | 0.146 | 139 | 0.123 | 115 | 1 |
| 4 | v1: Entropy rate \| v2: L2 proportion |  | z: Speaker population size |  |  |
|  | 0.113 | 52 | -0.005 | 52 |  |
| 5 | v1: Entropy rate \| v2: Speaker population size |  | z: L2 proportion |  |  |
|  | 0.365* | 67 | 0.291 | 52 |  |
| 6 | v1: Morphological complexity \| v2: Entropy rate |  |  |  |  |
|  | 0.127 | 39 |  |  | 1 |
| Area: S-Africa |  |  |  |  |  |
| 1 | v1: Speaker population size \| v2: L2 proportion |  |  |  |  |
|  | 0.472** | 134 |  |  |  |
| 2 | v1: Morphological complexity \| v2: L2 proportion |  | z: Speaker population size |  |  |
|  | -0.045 | 128 | -0.024 | 128 | 1 |
| 3 | v1: Morphological complexity \| v2: Speaker population size |  | z: L2 proportion |  |  |
|  | -0.032 | 134 | -0.029 | 128 | 1 |
| 4 | v1: Entropy rate \| v2: L2 proportion |  | z: Speaker population size |  |  |
|  | -0.129 | 38 | -0.239 | 38 |  |
| 5 | v1: Entropy rate \| v2: Speaker population size |  | z: L2 proportion |  |  |
|  | 0.246 | 44 | 0.268 | 38 |  |
| 6 | v1: Morphological complexity \| v2: Entropy rate |  |  |  |  |
|  | 0.010 | 36 |  |  | 1 |

Cf. Table 2 (main text) for a description of the columns. Here, separate analyses have been conducted for six language families and six geographical language areas. *p*-values are *Bonferroni* adjusted per family/area [*m* = 10].

# Section 5. Validation of the permutation test

In order to demonstrate the validity of the permutation test, population size (logged) is replaced by a randomly generated variable *v* where (i) the correlation between *v* and the dependent variable is (approximately) equal to the correlation between the dependent variable and the original population size variable, but (ii) the correlation between *v* and the original population size variable is (approximately) equal to zero. The approach is outlined here [7]. The permutation test is then repeated. If the test works, then we can expect the inclusion of the random variable as a (fixed) control variable to not affect the significance of the *vehicularity* variable. Table 5 demonstrates that it is indeed the case: in all but one models, *vehicularity* (at *p* < .01) significantly predicts morphological / information-theoretic complexity. This result suggests that it is indeed the population size that explains the apparent relationship between *vehicularity* and complexity (morphological/information-theoretic).

Table 5: Results of the validation of the permutation test

| Dependent variable | Control variable (fixed) | Control variable (random) | *R^2^* | Direction | *N* | *N_F_* |
| --- | --- | --- | --- | --- | --- | --- |
| Morphological complexity | no control |  | 1.38** | - | 1,581 | 1 |
|  | *v* |  | 1.49** | - | 1,581 |  |
|  |  | Families | 0.92** | - | 1,581 |  |
|  |  | Areas | 0.70* | - | 1,512 |  |
|  |  | Families & Areas | 0.64* | - | 1,512 |  |
|  |  | Families (intercepts & slopes) | 1.05** | - | 1,291 |  |
|  |  | Areas (intercepts & slopes) | 0.76* | - | 1,512 |  |
|  | no control |  | 1.92** | - | 862 | 6 |
|  | *v* |  | 1.89** | - | 862 |  |
|  |  | Families | 0.79* | - | 862 |  |
|  |  | Areas | 0.97* | - | 821 |  |
|  |  | Families & Areas | 0.79* | - | 821 |  |
|  |  | Families (intercepts & slopes) | 0.84 | - | 654 |  |
|  |  | Areas (intercepts & slopes) | 1.03* | - | 809 |  |
| Entropy rate | no control |  | 14.68** | + | 1,088 |  |
|  | *v* |  | 18.98** | + | 1,088 |  |
|  |  | Families | 3.21** | + | 1,088 |  |
|  |  | Areas | 4.73** | + | 719 |  |
|  |  | Families & Areas | 4.71** | + | 719 |  |
|  |  | Families (intercepts & slopes) | 3.65** | + | 912 |  |
|  |  | Areas (intercepts & slopes) | 5.44** | + | 695 |  |

Cf. Table 1 (main text) for a description of the columns. Here, in order to validate the permutation test, population size (logged) is replaced by a randomly generated variable *v*, where (i) the correlation between *v* and the dependent variable is (approximately) equal to the correlation between the dependent variable and the original population size variable, but (ii) the correlation between *v* and the original population size variable is (approximately) equal to zero.

# Section 6. Results of an alternative permutation test

Here, I use a generic version of Freedman & Lane’s permutation test [8]. The basic procedure is outlined in [9]:

1. Let *Y* denote the complexity (morphological/information-theoretic) variable. *Y* is regressed onto the log of population size and *vehicularity* in order to calculate the test statistic, here the *t*-statistic of the estimated parameter for *vehicularity* and call this statistic *T*_0_*.*
2. Complexity (morphological/information-theoretic) is regressed onto the log of population size *only* and fitted values $\hat{y}$ and residuals $\hat{\varepsilon}$ are obtained.
3. The residuals $\hat{\varepsilon}$ are randomly permuted, call the resulting variable $\hat{\varepsilon}^{*}$ and a new variable is computed that is defined as the sum of the fitted values and the randomly permuted residuals, i.e. $Y^{*}= \hat{y}+$ $\hat{\varepsilon}^{*}$.
4. $Y^{*}$is regressed onto the log of population size and *vehicularity* in order to calculate the test statistic of interest and call this statistic $T_{j}^{*}$*.*
5. Step 1 to 4 are repeated 100,000 times to calculate the reference distribution of $T^{*}$.
6. Count the number of times where ${|T}_{j}^{*}|\geq|T_{o}|$ and divide that number by 100,000. The result is the *p*-value.

The intuitive idea of this permutation test is that if the null hypothesis holds, i.e. there is no difference between *vehicular* and *non-vehicular* languages, the derived data sets, i.e. the data sets with randomly permuted residuals, should be equal to the original, or as [10; p.292] call it: “a small reported significance level indicates an unusual data set”. Table 6 demonstrates that this permutation test comes to the same conclusion as the test presented in the main part of the paper, as significance (at *p* < .01) is achieved in none of the models. Therefore, we cannot say that the influence of *vehicularity* is *substantial* enough to warrant the conclusion that large proportions of *L2* speakers affect the morphological and statistical structure of languages.

Table 6: Results of the alternative permutation test

| Dependent variable | Control variable (fixed) | Control variable (random) | *p*-value | *N* | *N_F_* |
| --- | --- | --- | --- | --- | --- |
| Entropy rate | Population size |  | 0.03 | 1,088 |  |
|  | Population size | Families | 0.49 | 1,088 |  |
| Unigram entropy | Population size |  | 0.14 | 1,080 |  |
|  | Population size | Families | 0.90 | 1,080 |  |
| Morphological complexity | Population size |  | 0.02 | 1,581 | 1 |
|  | Population size |  | 0.11 | 862 | 6 |
|  | Population size | Families | 0.02 | 1,581 | 1 |
|  | Population size | Families | 0.11 | 862 | 6 |

Table 5: 1st column: dependent variable. 2nd column: control variable (fixed). 3rd column: control variable (random). 4th column: *p*-value of the permutation test. 5th column: number of available languages. 7th column: number of included WALS features/chapters (if relevant). *NB*.: The population size is logged in all models. Values are rounded for illustration purposes only. One asterisks (*) indicates that the corresponding coefficient passed the permutation test at *p* < .01. Two asterisks (**) denote statistical significance after the Bonferroni adjustment [*m*  = 8].

# Section 7. Testing without languages that are categorized as *non-vehicular* languages but have *L2* proportions greater than zero

*Ethnologue* asserts that for *non-vehicular* languages, “*L2* users are not expected” [11]. However, there are a total of 78 *non-vehicular* languages for which Ethnologue reports an *L2* proportion greater than 0 (with a median estimate of 0.086). To rule out the possibility that those exceptions to the rule in the *Ethnologue* categorization scheme affect the results, separate analyses in which those 78 languages are dropped are presented in

Table 7 and Table 8. The results generally support the results presented in the main part of the paper. Compared to Table 2 in the main part of the paper, the only qualitative difference is row 2 of Table 8. It shows that there is a weak but significant negative correlation between morphological complexity and the *L2* proportion. However, when controlling for the speaker population size, the correlation strength is sharply reduced (both absolute values are below 0.1) and only the correlation for the full dataset (at least one available *WALS* feature) is significant. In addition, both part correlation coefficients do not reach statistical significance after the *Bonferroni* adjustment.

Table 7: Results of the permutation test

| Dependent variable | Control variable (fixed) | Control variable (random) | *R^2^* | Direction | *N* | *N_F_* |
| --- | --- | --- | --- | --- | --- | --- |
| Morphological complexity | no control |  | 1.32** | - | 1,513 | 1 |
|  | Population size |  | 0.17 | - | 1,513 |  |
|  |  | Families | 0.15 | - | 1,513 |  |
|  |  | Areas | 0.07 | - | 1,445 |  |
|  |  | Families & Areas | 0.06 | - | 1,445 |  |
|  |  | Families (intercepts & slopes) | 0.24 | - | 1,223 |  |
|  |  | Areas (intercepts & slopes) | 0.07 | - | 1,445 |  |
|  | no control |  | 1.79** | - | 825 | 6 |
|  | Population size |  | 0.09 | - | 825 |  |
|  |  | Families | 0.09 | - | 825 |  |
|  |  | Areas | 0.10 | - | 785 |  |
|  |  | Families & Areas | 0.09 | - | 785 |  |
|  |  | Families (intercepts & slopes) | 0.10 | - | 624 |  |
|  |  | Areas (intercepts & slopes) | 0.10 | - | 755 |  |
| Entropy rate | no control |  | 17.30** | + | 1,067 |  |
|  | Population size |  | 0.28 | + | 1,088 |  |
|  |  | Families | 0.03 | + | 1,088 |  |
|  |  | Areas | 0.01 | - | 719 |  |
|  |  | Families & Areas | 0.00 | - | 719 |  |
|  |  | Families (intercepts & slopes) | 0.00 | - | 912 |  |
|  |  | Areas (intercepts & slopes) | 0.03 | - | 695 |  |

Cf. Table 1 (main text) for a description of the columns.

Table 8: Summary of the correlation analysis.

| Row | *r*_v1v2_ | *N_r_* | *pr*_v1v2z_ | *N_r_* | *N_F_* |
| --- | --- | --- | --- | --- | --- |
| 1 | *v*_1_: Speaker population size \| *v*_2_: *L2* proportion |  |  |  |  |
|  | 0.309** | 1,913 |  |  |  |
| 2 | *v*_1_: Morphological complexity \| *v_2_*: *L2* proportion |  | *z*: Speaker population size |  |  |
|  | -0.106** | 1,382 | -0.065* | 1,382 | 1 |
|  | -0.135** | 737 | -0.060 | 737 | 6 |
| 3 | *v*_1_: Morphological complexity \| *v_2_*: Speaker population size |  | *z*: *L2* proportion |  |  |
|  | -0.137** | 1,513 | -0.096** | 1,382 | 1 |
|  | -0.179** | 825 | -0.144** | 737 | 6 |
| 4 | *v*_1_: Entropy rate \| *v_2_*: *L2* proportion |  | *z*: Speaker population size |  |  |
|  | 0.278** | 965 | 0.086* | 965 |  |
| 5 | *v*_1_: Entropy rate \| *v_2_*: Speaker population size |  | z: *L2* proportion |  |  |
|  | 0.561** | 1,067 | 0.433** | 965 |  |
| 6 | *v*_1_: Morphological complexity \| *v_2_*: Entropy rate |  |  |  |  |
|  | -0.015 | 515 |  |  | 1 |
|  | 0.047 | 329 |  |  | 6 |

Cf. Table 2 (main text) for a description of the columns.

.

# Section 8. Using the total number of speakers instead of the number of L1 speakers

As described in the main part of the paper in the Material and Methods section, the number of *L1* speakers is used as a measure of speaker population size. Here, I present additional analyses that use the total number of speakers; it can be calculated as:

$$N_{Total}=\frac{N_{L1}}{1-p_{L2}} \mathrm{if} p_{L2}\neq1$$

where $p_{L2}$ is the estimated *L2* proportion and $N_{L1}$ is the estimated number of *L1* speakers. Note that there are three languages in the data (Clallam, Cornish and Klamath-Modoc) that have an $N_{L1}$ that is greater than zero according to the used data provided by [12]. However, for those three languages, the 20th edition of the Ethnologue [13] lists an estimated number of *L1* speakers that is equal to zero. For those three languages, the $N_{L1}$ estimate provided by [12] is used as $N_{Total}$. Languages without an available *L2* proportion estimate (*N*  = 152) are not used for the analyses presented in this section.

Table 9 and Table 10 demonstrate that the results presented in Table 1 and Table 2 of the main part of the paper are not qualitatively affected if the total number of speakers is used instead of the number of *L1* speakers.

Table 9: Results of the permutation test

| Dependent variable | Control variable (fixed) | Control variable (random) | *R^2^* | Direction | *N* | *N_F_* |
| --- | --- | --- | --- | --- | --- | --- |
| Morphological complexity | no control |  | 0.95** | - | 1,450 | 1 |
|  | Population size |  | 0.18 | - | 1,450 |  |
|  |  | Families | 0.23 | - | 1,450 |  |
|  |  | Areas | 0.22 | - | 1,389 |  |
|  |  | Families & Areas | 0.22 | - | 1,389 |  |
|  |  | Families (intercepts & slopes) | 0.24 | - | 1,158 |  |
|  |  | Areas (intercepts & slopes) | 0.21 | - | 1,389 |  |
|  | no control |  | 1.65** | - | 774 | 6 |
|  | Population size |  | 0.11 | - | 774 |  |
|  |  | Families | 0.20 | - | 774 |  |
|  |  | Areas | 0.34 | - | 739 |  |
|  |  | Families & Areas | 0.38 | - | 739 |  |
|  |  | Families (intercepts & slopes) | 0.17 | - | 571 |  |
|  |  | Areas (intercepts & slopes) | 0.27 | - | 719 |  |
| Entropy rate | no control |  | 9.54** | + | 986 |  |
|  | Population size |  | 0.01 | + | 986 |  |
|  |  | Families | 0.00 | - | 986 |  |
|  |  | Areas | 0.02 | - | 629 |  |
|  |  | Families & Areas | 0.08 | - | 629 |  |
|  |  | Families (intercepts & slopes) | 0.04 | - | 821 |  |
|  |  | Areas (intercepts & slopes) | 0.06 | - | 603 |  |

Cf. Table 1 (main text) for a description of the columns.

Table 10: Summary of the correlation analysis.

| Row | *r*_v1v2_ | *N_r_* | *pr*_v1v2z_ | *N_r_* | *N_F_* |
| --- | --- | --- | --- | --- | --- |
| 1 | *v*_1_: Speaker population size \| *v*_2_: *L2* proportion |  |  |  |  |
|  | 0.282** | 1,991 |  |  |  |
| 2 | *v*_1_: Morphological complexity \| *v_2_*: *L2* proportion |  | *z*: Speaker population size |  |  |
|  | -0.044 | 1,450 | -0.011 | 1,382 | 1 |
|  | -0.066 | 774 | -0.003 | 737 | 6 |
| 3 | *v*_1_: Morphological complexity \| *v_2_*: Speaker population size |  | *z*: *L2* proportion |  |  |
|  | -0.112** | 1,450 | -0.104** | 1,382 | 1 |
|  | -0.171** | 774 | -0.158** | 737 | 6 |
| 4 | *v*_1_: Entropy rate \| *v_2_*: *L2* proportion |  | *z*: Speaker population size |  |  |
|  | 0.295** | 986 | 0.075* | 986 |  |
| 5 | *v*_1_: Entropy rate \| *v_2_*: Speaker population size |  | z: *L2* proportion |  |  |
|  | 0.510** | 986 | 0.423** | 986 |  |
| 6 | *v*_1_: Morphological complexity \| *v_2_*: Entropy rate |  |  |  |  |
|  | 0.011 | 445 |  |  | 1 |
|  | 0.028 | 275 |  |  | 6 |

Cf. Table 2 (main text) for a description of the columns.

# Section 9. Binary mediation analysis

One anonymous reviewer suggested a mediation analysis as an alternative way of testing the linguistic niche hypothesis. In this section, I present the results of a binary mediation analysis [14] in which the question whether *vehicularity* mediates the association between complexity (morphological/information-theoretic) and speaker population size (logged) is tested.

The basic procedure is as follows:

1. A logistic regression of *vehicularity* on the log of speaker population size is conducted.
2. An ordinary least squares regression of complexity (morphological/information-theoretic) on the log of speaker population size is conducted.
3. An ordinary least squares regression of complexity (morphological/information-theoretic) on *vehicularity* on the log of speaker population size is conducted.
4. Indirect effects are computed as the product of rescaled (i.e. standaridzed) coefficients.
5. Bias corrected confidence intervals (99%) are calculated by bootstraping with 10,000 replications

Table 11 demonstrates that the confidence interval includes zero in all three tests. This indicates that the indirect effect is not significantly different from zero. Therefore, the mediated proportion is essentially meaningless, so we cannot say that *vehicularity* mediates the association between complexity and speaker population size.

Table 11: Summary of the binary mediation analysis.

| Dependent variable | Independent variable | Mediator variable | Proportion of total effect mediated | 99% Conf. Interval for the total indirect effect | | *N* | *N_F_* |
| --- | --- | --- | --- | --- | --- | --- | --- |
| Morphological complexity | Population size | vehicularity | .0883 | -.1152 | .0051 | 1,581 | 1 |
|  |  |  | .2861 | -.1394 | .0395 | 862 | 6 |
| Entropy rate |  |  | .0514 | -.0159 | .1185 | 1,088 |  |

1st column: dependent variable. 2nd column: independent variable. 3rd column: mediator variable. 4th column: Proportion of total effect mediated. 5th and 6th column: 99% confidence interval for the total indirect effect. 7^th^ column: number of available languages. 7th column: number of included WALS features/chapters (if relevant). *NB*.: The population size is logged in all models. Values are rounded for illustration purposes only. Bias corrected confidence intervals are calculated by bootstraping with 10,000 replications.

# Section 10. Testing only for languages that are categorized as *vehicular*

In this section, I present an additional correlation analysis that only includes languages that are categorized as *vehicular*. Table 12 presents the results. Again, the results generally support the results presented in the main part of the paper.

Compared to Table 2 in the main part of the paper, the main differences are:

1. A rather surprising negative correlation between speaker population size and the proportion of *L2* speakers (cf. row 1).
2. A negative correlation between the entropy rate and the proportion of *L2* speakers (cf. row 4). However, when the effect of the speaker population size is removed, the resulting correlation coefficient is sharply reduced and does not pass the permutation test.
3. A significant but positive correlation between morphological complexity and the entropy rate for *N*_F_ ≥ 6 (cf. row 6).

As written in the main part of the paper, for both (i), (ii) and (iii), the linguistic niche hypothesis predicts an association that should run in the opposite direction. Apart from those points, this analysis does not support the linguistic niche hypothesis, because there is no significant negative correlation between morphological complexity and the *L2* proportion, neither for *N*_F_ ≥ 1, nor for *N*_F_ ≥ 6. The entropy rate correlates significantly with the *L2* proportion (cf. row 4); however when the effect of the speaker population size is removed, the resulting correlation coefficient is sharply reduced and does not pass the permutation test. In accordance with the other results presented in this paper, row 5 reveals that there is a strong and significant positive correlation between the entropy rate and the estimated speaker population size.

Table 12: Summary of the correlation analysis.

| Row | *r*_v1v2_ | *N_r_* | *pr*_v1v2z_ | *N_r_* | *N_F_* |
| --- | --- | --- | --- | --- | --- |
| 1 | *v*_1_: Speaker population size \| *v*_2_: *L2* proportion |  |  |  |  |
|  | -0.391** | 89 |  |  |  |
| 2 | *v*_1_: Morphological complexity \| *v_2_*: *L2* proportion |  | *z*: Speaker population size |  |  |
|  | -0.228 | 76 | -0.246 | 76 | 1 |
|  | -0.226 | 60 | -0.235 | 60 | 6 |
| 3 | *v*_1_: Morphological complexity \| *v_2_*: Speaker population size |  | *z*: *L2* proportion |  |  |
|  | 0.043 | 207 | -0.092 | 76 | 1 |
|  | 0.116 | 148 | -0.065 | 60 | 6 |
| 4 | *v*_1_: Entropy rate \| *v_2_*: *L2* proportion |  | *z*: Speaker population size |  |  |
|  | -0.320* | 73 | -0.079 | 73 |  |
| 5 | *v*_1_: Entropy rate \| *v_2_*: Speaker population size |  | z: *L2* proportion |  |  |
|  | 0.517** | 175 | 0.527** | 73 |  |
| 6 | *v*_1_: Morphological complexity \| *v_2_*: Entropy rate |  |  |  |  |
|  | 0.097 | 141 |  |  | 1 |
|  | 0.307** | 109 |  |  | 6 |

Cf. Table 2 (main text) for a description of the columns.

# References

1. Jaeger TF, Graff P, Croft W, Pontillo D. 2011 Mixed effect models for genetic and areal dependencies in linguistic typology. *Linguistic Typology* **15**. (doi:10.1515/lity.2011.021)

2. Bentz C, Verkerk A, Kiela D, Hill F, Buttery P. 2015 Adaptive Communication: Languages with More Non-Native Speakers Tend to Have Fewer Word Forms. *PLOS ONE* **10**, e0128254. (doi:10.1371/journal.pone.0128254)

3. Bentz C, Winter B. 2013 Languages with More Second Language Learners Tend to Lose Nominal Case. *Language Dynamics and Change* **3**, 1–27. (doi:https://doi.org/10.1163/22105832-13030105)

4. Lupyan G, Dale R. 2010 Language Structure Is Partly Determined by Social Structure. *PLoS ONE* **5**, e8559. (doi:10.1371/journal.pone.0008559)

5. Bentz C, Alikaniotis D, Cysouw M, Ferrer-i-Cancho R. 2017 The Entropy of Words—Learnability and Expressivity across More than 1000 Languages. *Entropy* **19**, 275. (doi:10.3390/e19060275)

6. Mayer T, Cysouw M. 2014 Creating a Massively Parallel Bible Corpus. In *Proceedings of the Ninth International Conference on Language Resources and Evaluation (LREC’14)* (eds NC (Conference Chair), K Choukri, T Declerck, H Loftsson, B Maegaard, J Mariani, A Moreno, J Odijk, S Piperidis), Reykjavik, Iceland: European Language Resources Association (ELRA).

7. Schechter C, Lacy M. 2014 Creating a variable with a known correlation with existing variables. *Statalist - The Stata Forum*.

8. Still AW, White AP. 1981 The approximate randomization test as an alternative to the F test in analysis of variance. *British Journal of Mathematical and Statistical Psychology* **34**, 243–252. (doi:10.1111/j.2044-8317.1981.tb00634.x)

9. Winkler AM, Ridgway GR, Webster MA, Smith SM, Nichols TE. 2014 Permutation inference for the general linear model. *NeuroImage* **92**, 381–397. (doi:10.1016/j.neuroimage.2014.01.060)

10. Freedman DA, Lane D. 1983 A Nonstochastic Interpretation of Reported Significance Levels. *Journal of Business & Economic Statistics* **1**, 292. (doi:10.2307/1391660)

11. Simons GF, Fennig CD. 2017 Ethnologue Global Dataset, Twentieth edition. See https://www.ethnologue.com/sites/default/files/Ethnologue-20-Global%20Dataset%20Doc.pdf.

12. Amano T, Sandel B, Eager H, Bulteau E, Svenning J-C, Dalsgaard B, Rahbek C, Davies RG, Sutherland WJ. 2014 Global distribution and drivers of language extinction risk. *Proceedings of the Royal Society B: Biological Sciences* **281**, 20141574–20141574. (doi:10.1098/rspb.2014.1574)

13. Simons, Gary F, Fennig CD. 2017 *Ethnologue: Languages of the World*. Dallas, Texas: SIL International.

14. Ender P. 2011 *binary_mediation: Stata module for mediation analysis with binary mediator and/or response variables*. UCLA: Statistical Consulting Group. See https://stats.idre.ucla.edu/stata/ado/analysis/.
